# The Influence of Extracellular Citrate in Physiological Concentration on the Proliferation of Malignant Melanoma

**DOI:** 10.1111/jcmm.71082

**Published:** 2026-03-02

**Authors:** Konstantin Drexler, Barbara Schwertner, Veronika Zenderowski, Laura Schreieder, Dennis Christoph Harrer, Mark Berneburg, Edward Geissler, Maria Mycielska, Sebastian Haferkamp

**Affiliations:** ^1^ Department of Dermatology University Hospital Regensburg Regensburg Germany; ^2^ Department of Internal Medicine III, Hematology and Oncology University Hospital Regensburg Regensburg Germany; ^3^ Department of Surgery University Hospital Regensburg Regensburg Germany; ^4^ Department of Structural Biology Institute of Biophysics and Physical Biochemistry, University of Regensburg Regensburg Germany

**Keywords:** citrate, gluconate, malignant melanoma, pmCiC

## Abstract

Cancer cells rely on citrate for multiple metabolic processes, suggesting that limiting the availability of extracellular citrate may represent a novel therapeutic strategy. The plasma membrane citrate transporter (pmCiC) has been implicated in the pathogenesis of several cancers before, and its activity can be inhibited by gluconate. Tissue samples from patients were stained, and pmCiC expression was analysed and correlated with clinical course. Melanoma cells were treated with or without citrate in physiological concentration and with or without gluconate, a pmCiC inhibitor. Cell proliferation rates were subsequently measured. pmCiC expression was observed in 58.2% of primary melanomas and 76.5% of melanoma metastases, but only in 22.2% of benign nevi. However, pmCiC expression did not correlate with the response to novel melanoma‐specific therapies. In the presence of pmCiC, melanoma cells exhibited significantly increased proliferation when exposed to extracellular citrate. This effect was blocked by the addition of gluconate. Extracellular citrate uptake via pmCiC appears to contribute to the pathogenesis of malignant melanoma. Notably, inhibition of pmCiC by gluconate effectively suppressed citrate‐induced proliferation.

## 1 Introduction

Malignant melanoma is responsible for over 90% of skin cancer‐related deaths, with tumour thickness closely correlating with aggressiveness and metastatic potential. While early‐stage prognosis is favourable, treating advanced disease remains challenging. Recent therapies, such as immune checkpoint inhibitors (anti‐PD‐1, anti‐CTLA‐4) and BRAF/MEK inhibitors, have improved survival, yet novel treatment strategies are still needed [1].

Tumour metabolism has emerged as a critical driver of cancer progression. Unlike normal cells, cancer cells reprogram their metabolism to support proliferation, survival and adaptation [2]. Citrate, a central molecule in the mitochondrial Krebs cycle and the primary substrate in fatty acid synthesis—a hallmark of cancer—plays a key role in energy production and biosynthesis of different metabolites [3, 4]. Recent studies suggest that cancer cells can manipulate extracellular citrate levels, influencing the tumour microenvironment. Low intracellular citrate can trigger stromal cells—such as cancer‐associated fibroblasts—to release more citrate, which is associated with metastatic behaviour [5, 6]. A plasma membrane variant of the mitochondrial citrate transporter, known as pmCiC, has been identified in several cancers, showing a percentage of positive vs. negative tumours in 55% of breast cancers, 54% of gastric cancers, 86% of pancreatic cancers, and 86% of merkel cell carcinoma, where it enables citrate uptake for anabolic processes like fatty acid synthesis [5, 7, 8]. pmCiC expression has been linked to tumour aggressiveness across different entities, like lung, prostate and urothelial cancer [9] and was recently shown to promote proliferation in Merkel cell carcinoma, where its inhibition by gluconate reduced tumour growth in vitro and in vivo [7, 10]. We have already shown that gluconate binds to the citrate‐binding site of pmCiC and effectively blocks citrate transport in a concentration of 100 μM [5, 7]. In this study, we explored the role of pmCiC in melanoma and assessed the effects of citrate in physiological concentration and its inhibitor gluconate on melanoma cell behaviour in vitro.

## 2 Methods

### 2.1 Immunohistochemistry

Paraffin‐embedded sections were dewaxed using xylol (Merck, Darmstadt, Germany) and then rehydrated by washing twice with absolute ethanol, twice with 96% ethanol, and twice with 70% ethanol. After blocking endogenous peroxidases using 3% H_2_O_2_ for 10 min, samples were washed once with bi‐distilled water, heated in HIER Citrate Buffer pH 6 (Zytomed/Biozol, Eching, Germany) at 90°C for 20 min, and cooled for 20 min. Samples were blocked for 10 min using the blocking solution of the ZytoChem Plus HRP‐kit Rab‐bit/Mouse (Zytomed). Sections were labelled at 4°C overnight using a rabbit monoclonal antibody against pmCiC (D2P2F, Cell Signalling; dilution for patient samples and for cell lines 1:200). After washing with DPBS, sections were incubated with biotinylated secondary anti‐rabbit (HRP060‐RB) antibodies for 30 min, washed in DPBS, and incubated with streptavidin HRP conjugate for 20 min (all from ZytoChem Plus HRP Kit, Zytomed). After washing with DPBS, slides were stained using AEC+ High Sensitivity Substrate Chromo‐gen Ready‐to‐Use (Dako/Agilent Technologies, Hamburg, Germany), counterstained using haematoxylin (Carl Roth, Karlsruhe, Germany), and mounted using Aquatex (Merck). Afterwards pmCiC expression was scored by four different investigators (Fleiss' kappa = 0.407; 95% CI: 0.353, 0.460). Tissues were collected from patients of the Department of Dermatology at the University Hospital Regensburg and correlated with clinical outcome; two groups of tissues were analysed. Ethics permission was given: #22–2834‐104 [7].

### 2.2 Cell Culture

A cell line from primary melanoma (IGR39) was grown in DMEM medium with 1% penicillin/streptomycin, 1% L‐glutamine and 10% fetal bovine serum. Although cells were not further authenticated, they were grown at low passage numbers from original sources and were kept typically in culture for only 2 months. Cells were tested and confirmed to be mycoplasma free. The following chemicals were used: citric acid and Na^+^‐gluconate (Sigma, St. Louis, MO, USA), and dialyzed serum (PAN Biotech GmbH, Aidenbach, Germany). The following antibodies were used: pmCiC specific antibodies (12; custom‐made by GenScript Inc., Piscataway, NJ, USA). Experimental media consisted of glucose‐free DMEM medium (Sigma, St. Louis, MO, USA), 10% dialyzed serum, 2 mM glutamine, 0.25 g/L glucose, ±200 μM citrate, and ±100 μM Na^+^ gluconate, unless otherwise stated. The incubation time varied between 24, 48, 72 h and 5 weeks, as specified.

### 2.3 Proliferation Assay

To measure proliferation, cells were incubated in a 96‐well plate and Incucyte live cell imaging by satrtorius (Sartorius AG, Göttingen, Germany) was used. Pictures of the cells were taken every 2 h and cell confluence was measured.

### 2.4 Statistics

Statistics were performed using GraphPad by Dotmatics and IBM SPSS version 25 (Armonk, NY, USA). Significance was assumed at *p* < 0.05.

## 3 Results

### 3.1 Expression of pmCiC in Malignant Melanoma

**FIGURE 1:**
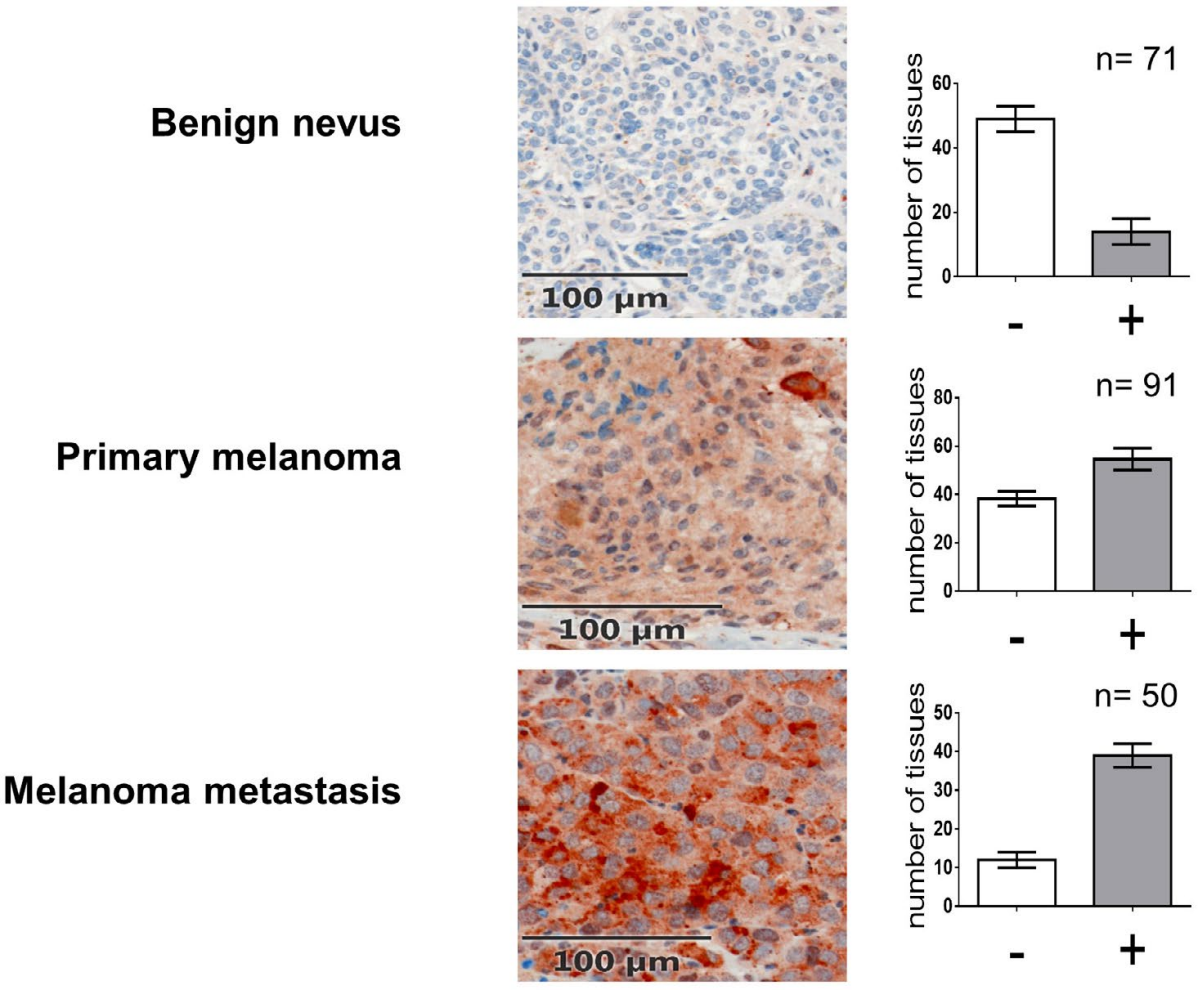
Expression of pmCiC is different in benign nevi, primary melanoma and melanoma metastasis.

**FIGURE 2:**
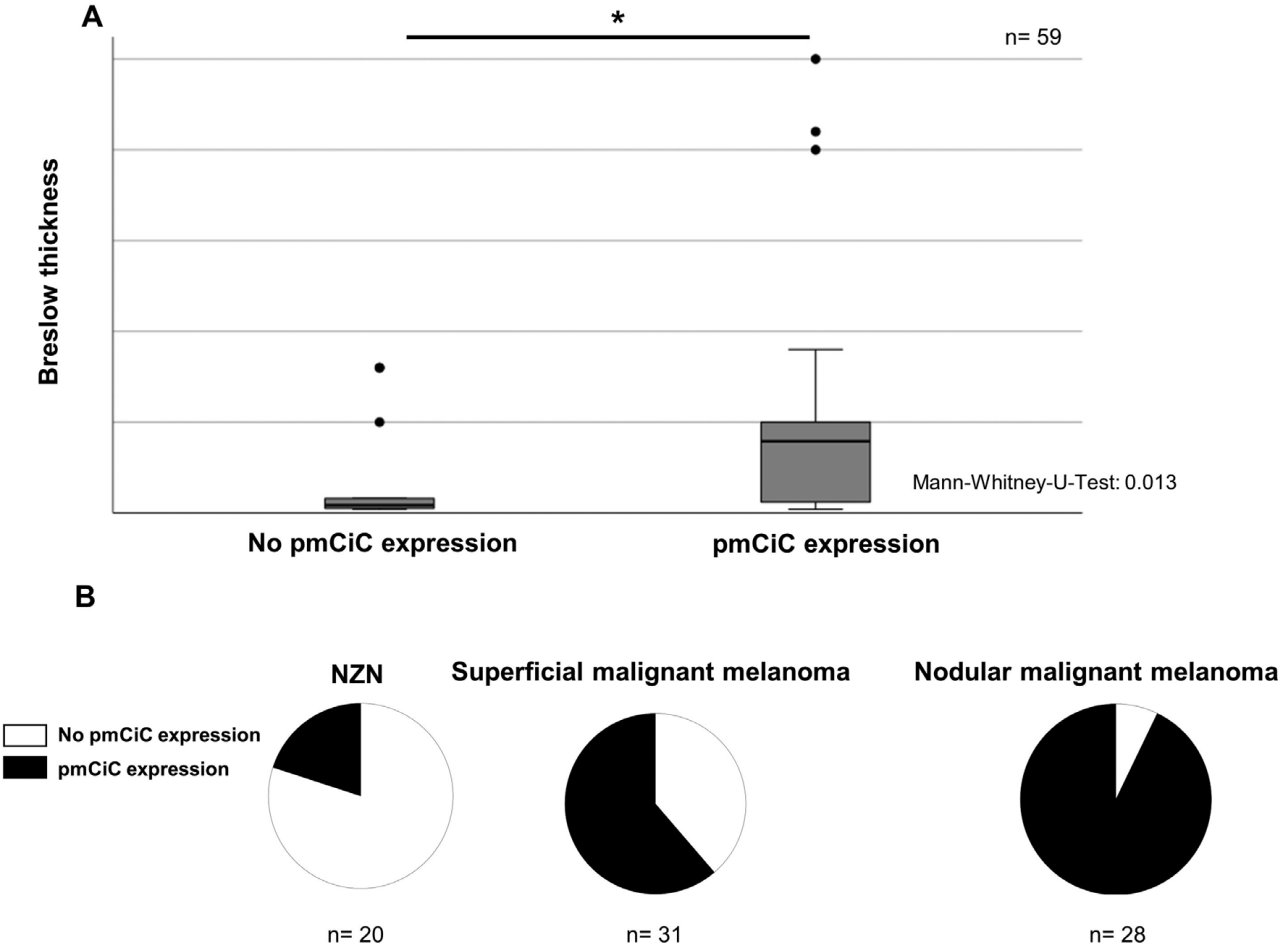
(A) Expression of pmCiC in primary melanoma is correlating with tumour thickness. (B) Expression of pmCiC in nevi and different histological subtypes of melanoma.

Immunohistochemical analysis was performed on tissue samples from primary melanomas, melanoma metastases and benign nevi. pmCiC expression was detected in 58.2% of primary melanomas and 76.5% of melanoma metastases, but only in 22.2% of benign nevi (Figure 1), indicating a correlation with increasing malignancy. In primary melanomas, higher pmCiC expression was significantly associated with greater tumour thickness (*p* = 0.013, Mann–Whitney‐U‐Test; Figure 2A). Among melanoma subtypes, nodular melanoma, known for its poorer prognosis, showed higher pmCiC expression than superficial spreading melanoma (Figure 2B).

To assess potential clinical relevance, baseline metastatic samples from stage IV melanoma patients were evaluated. pmCiC expression at baseline did not differ between responders (*n* = 42) and non‐responders (*n* = 43; *p* = 0.447) to immune checkpoint inhibitors or BRAF/MEK inhibitors. Hence, pmCiC expression in metastases was not predictive of treatment response or clinical outcome in advanced disease.

### 3.2 Expression of pmCiC in Vitro

**FIGURE 3:**
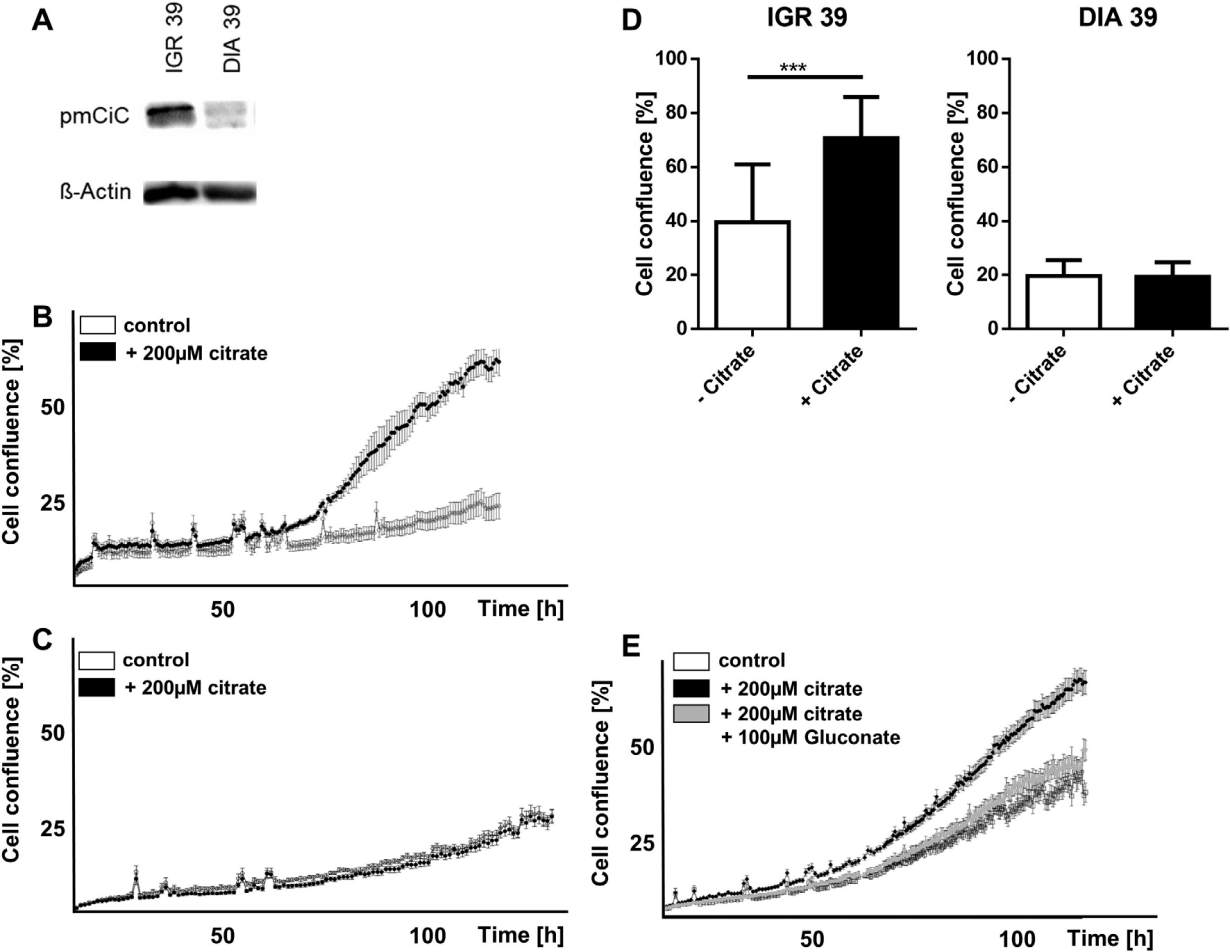
(A) Western blot analysis showing expression of pmCiC in different melanoma cell lines. (B) Cell confluence of melanoma cells IGR 39 depending on the presence of citrate. (C) Cell confluence of DIA 39 melanoma cells, depending on the presence of citrate. (D) Comparison of the influence of citrate on the proliferation rate, depending on the presence of pmCiC. (E) Cell confluence of IGR 39 melanoma cells, depending on the presence of citrate and the citrate inhibitor, gluconate.

To further explore the role of pmCiC in melanoma, its expression was analysed across a panel of melanoma and melanocyte cell lines. The results revealed substantial variability among melanoma cell lines, with some exhibiting high levels of pmCiC, while others showed moderate, low, or no detectable expression. In contrast, pmCiC expression was absent or only minimally present in non‐malignant melanocyte cell lines, suggesting that upregulation of pmCiC may be associated with malignant transformation. Based on these findings, two representative melanoma cell lines were selected for further functional studies: IGR 39, which showed high pmCiC expression, and DIA 39 which lacked detectable expression. These two models were used to investigate the potential role of pmCiC in melanoma cell behaviour under different experimental conditions (Figure 3A).

### 3.3 Effect of Extracellular Citrate on Melanoma Cell Proliferation

To assess the impact of extracellular citrate on melanoma cell proliferation, cells were incubated with or without citrate, and proliferation was monitored using the Incucyte assay. A dose‐dependent increase in proliferation was observed at citrate concentrations between 50 μM and 2000 μM (Figure S1), while higher dosage did not show any positive effects (most likely due to Ca^2+^ chelation). Based on previous studies [11] and physiological serum levels, 200 μM was selected for subsequent experiments. Herein, the high‐expressing IGR 39 melanoma cell line showed significantly increased proliferation in the presence of extracellular citrate (Figure 3B,D). In contrast, no such effect was observed in the non‐pmCiC expressing DIA 39 melanoma cell line (Figure 3C,D). Both cell lines were tested for the expression of the sodium‐dependent transporter NaCT (SCL13A5) showing no differences in western blot analysis (Figure S1). This suggests that citrate‐induced proliferation depends on the presence of pmCiC.

### 3.4 Inhibition of Citrate‐Induced Proliferation by Gluconate

Previous studies identified gluconate as a potent inhibitor of pmCiC [11]. To confirm the role of pmCiC in citrate‐induced proliferation, sodium or calcium gluconate was added to melanoma cells, and proliferation was reassessed using the Incucyte assay. In strongly‐pmCiC expressing IGR 39 cells, the proliferative effect of extracellular citrate was effectively blocked by gluconate (Figure 3E). pH measurements confirmed that this effect was not due to changes in medium acidity (Figure S1). No impact of citrate was observed on cell migration or invasion, as shown by scratch and invasion assays (Figure S1).

## 4 Discussion

This study demonstrates that pmCiC is expressed in malignant melanoma and may contribute to tumour progression by enabling the uptake of extracellular citrate for metabolic use. pmCiC was detected in 58.2% of primary melanomas and 76.5% of metastases, but only in 22.2% of benign nevi, suggesting progressively increased involvement as tumours reach a more advanced stage. This pattern implies that melanoma cells, unlike benign melanocytes, may exploit extracellular citrate to support anabolic and proliferative processes. A similar association between metabolite transport and tumour aggressiveness has been reported for Monocarboxylate Transporter 1 (MCT1), which facilitates lactate uptake and is linked to metastasis in melanoma [12]. Differences in transporter expression levels among individual melanomas are also known for other membrane transporters, depending on subtype and aggressiveness [13]. Interestingly, 22% of benign nevi also showed an expression of pmCiC. We also observed an expression of the transporter in other benign cells, like cancer associated fibroblasts before, which needs some further investigations [5]. According to our hypothesis of the importance of citrate for tumour proliferation and prognosis, Yang et al. described a correlation of prognosis and an upregulation of SLC13A5 in the tumour microenvironment in melanoma patients [14]. In our study, a direct correlation between pmCiC expression and citrate‐induced proliferation was observed. Based on the collected data, extracellular citrate uptake might play a significant role in promoting proliferation by making cancer cell metabolism more efficient, as shown previously [11]. The proliferative effect of extracellular citrate in physiological concentration was abolished by sodium gluconate, a known pmCiC inhibitor, confirming the transporter's potential functional relevance. Gluconate alone showed no effect on proliferation of tumour cells in previous studies [7, 11].

Notably, melanoma thickness remains a strong prognostic factor and correlates with pmCiC expression like other diagnostic markers, such as PRAME, which also correlate with clinical outcomes [15]. In our experiments, extracellular citrate at physiological concentrations promoted melanoma cell proliferation, whereas higher concentrations (e.g., 5000 μM) abolished this effect, consistent with findings by Zhao et al. [16]. This concentration‐dependent effect may be due to citrate's acidity, as high extracellular acidity can be cytotoxic [17] or potentially related to Ca^2+^ chelation [18]. Interestingly, cancer cells generally favour mildly acidic environments (pH ~6.5), which promote tumour growth and immune evasion [19].

Interestingly, the proliferative effect of extracellular citrate in physiological concentration was blocked by gluconate, as we have previously reported [7]. Gluconate, a non‐toxic substance used in medicine for its cation‐binding properties, cannot cross the plasma membrane itself [10, 20, 21]. While gluconate could therefore be considered therapeutically in the future, it would be necessary to better understand the different off‐target cell types affected and the mechanism by which it inhibits pmCiC. Also, the pharmacological properties of gluconate should be part of further studies to investigate how it could be used in the therapy of cancer patients. Further studies are needed to answer this question.

In a broader view in oncology, citrate appears to be imported via pmCiC not only in melanoma but also in other tumour types, like breast, gastric, pancreatic, lung, prostate and urothelial cancer as well as merkel cell carcinoma. This supports the hypothesis that cancer cells express a variant of the mitochondrial citrate transporter in the plasma membrane to enhance citrate uptake [5, 7, 11]. Similar investigations will be needed to confirm the role of pmCiC in other cancer types.

## 5 Conclusion

This study is the first to demonstrate high expression levels of pmCiC in progressively malignant melanoma cells and to explore its association with tumour behaviour and patient prognosis. We also provide initial evidence that extracellular citrate promotes melanoma cell proliferation in a pmCiC‐dependent manner. This proliferative effect was effectively inhibited by gluconate in vitro, highlighting its potential as a candidate for further investigation as an antitumor agent.

## Author Contributions

Konstantin Drexler, Maria Mycielska and Sebastian Haferkamp: conceptualization. Konstantin Drexler, Barbara Schwertner, Veronika Zenderowski and Laura Schreieder: data curation. Konstantin Drexler; Investigation, Konstantin Drexler: formal analysis. Konstantin Drexler, Barbara Schwertner, Maria Mycielska, Sebastian Haferkamp: methodology. Konstantin Drexler, Mark Berneburg and Sebastian Haferkamp: Resources. Konstantin Drexler: software. Maria Mycielska, Edward Geissler and Sebastian Haferkamp: supervision. Dennis Christoph Harrer and Edward Geissler: validation. Konstantin Drexler: visualisation. Konstantin Drexler: writing – original draft. Konstantin Drexler, Barbara Schwertner, Veronika Zenderowski, Laura Schreieder, Dennis Christoph Harrer, Mark Berneburg, Maria Mycielska, Edward Geissler, Sebastian Haferkamp: writing – review and editing.

## Funding

This work was supported by Else Kröner‐Fresenius‐Stiftung and Bayerisches Zentrum für Krebsforschung (BZKF).

## Ethics Statement

Ethics permission was given by the University of Regensburg: #22‐2834‐104.

## Conflicts of Interest

The authors declare no conflicts of interest.

## Supporting information


**Figure S1:** (A) Proliferation of IGR 39 melanoma cells, depending on the concentration of extracellular citrate. (B) pH measurements showing no differences after adding 200 μM citrate. (C) Extracellular citrate has no effects on invasion and scratch assays in the IGR 39 melanoma cell line. (D) Western blot analysis showing no differences in the expression of citrate transporter SLC13A5 in the investigated cell lines (IGR39 and 3 replicates of DIA39).

## Data Availability

The data that support the findings of this study are available on request from the corresponding author. The data are not publicly available due to privacy or ethical restrictions.
