# Supplementary figures and images for: The Influence of Extracellular Citrate in Physiological Concentration on the Proliferation of Malignant Melanoma
Source: J Cell Mol Med. 2026 Mar 2;30(5):e71082. doi: 10.1111/jcmm.71082 (PMC12953189; doi:10.1111/jcmm.71082)

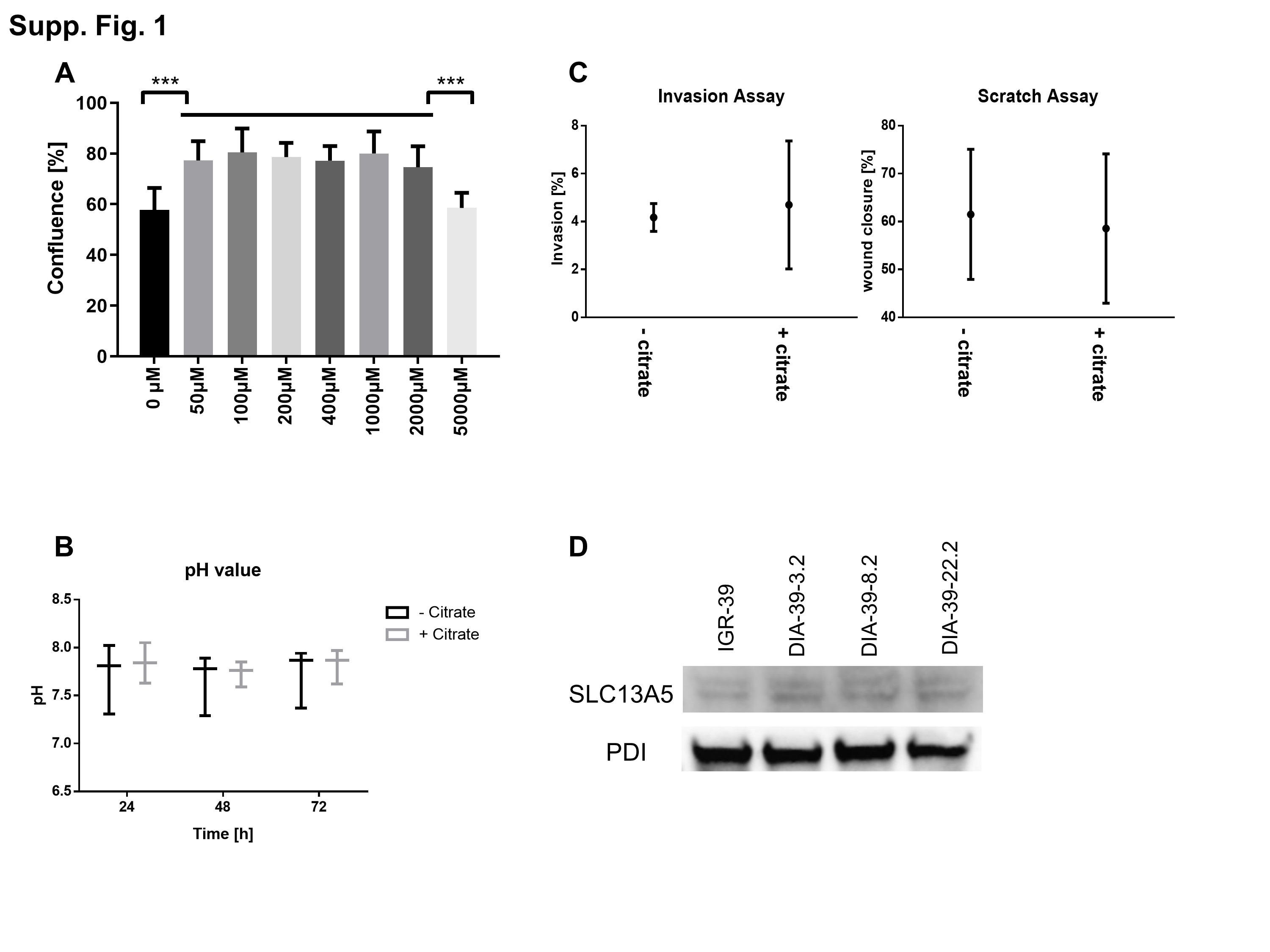

Supplement: Supplementary file 1 — Figure S1: (A) Proliferation of IGR 39 melanoma cells, depending on the concentration of extracellular citrate. (B) pH measurements showing no differences after adding 200 μM citrate. (C) Extracellular citrate has no effects on invasion and scratch assays in the IGR 39 melanoma cell line. (D) Western blot analysis showing no differences in the expression of citrate transporter SLC13A5 in the investigated cell lines (IGR39 and 3 replicates of DIA39). [file JCMM-30-e71082-s001.jpg]
